# The Logic of Interactive Biorobotics

**DOI:** 10.3389/fbioe.2020.00637

**Published:** 2020-07-08

**Authors:** Edoardo Datteri

**Affiliations:** RobotiCSS Lab - Laboratory of Robotics for the Cognitive and Social Sciences, Department of Human Sciences for Education, University of Milano-Bicocca, Milan, Italy

**Keywords:** interactive robotics, biorobotics, ethorobotics, robot-animal interaction, biologically inspired robotics

## Abstract

In recent studies, robots are used to stimulate living systems in controlled experimental settings. This research strategy is here called interactive biorobotics, to distinguish it from classical biorobotics, in which robots are used to simulate, rather than to stimulate, living system behavior. This article offers a methodological analysis of interactive biorobotics and has two goals. The first one is to argue that interactive biorobotics is methodologically different, in some important respects, from classical biorobotics and from countless instances of model-based science. It will be shown that interactive biorobotics does not conform to the so-called “understanding by building” approach or synthetic method, and that it illustrates a novel use of models in science. The second goal is to reflect on the logic of interactive biorobotics. A distinction will be made between two classes of studies, which will be called “proximal” and “distal.” In proximal studies, experiments involving robot-animal interaction are brought to bear on theoretical hypotheses on robot-animal interaction. In distal studies, experiments involving robot-animal interaction are brought to bear on theoretical hypotheses on animal-animal interaction. Distal studies involve logical steps which may be particularly hard to justify. This distinction, together with a methodological reflection on the relationship between the context in which the experiments are carried out and the context in which the conclusions are expected to hold, will lead to a checklist of questions which may be useful to justify and evaluate the validity of interactive biorobotics studies. The reconstruction of the logic of interactive biorobotics made here, though preliminary, may contribute to justifying the important role that robots, as tool for stimulating living systems, can play in the contemporary life sciences.

## Introduction

Automata have inspired the formulation of theories on animal and human behavior for centuries. The flourishing of “clocks, artificial fountains, mills, and other such machines which, although only man-made, have the power to move of their own accord in many different ways” (Descartes, 1664/1985) inspired the development of mechanistic conceptions of life and animal behavior in the modern age (Riskin, 2016). de La Mettrie, author of the treatise *L'homme machine* (1747/1960), was familiar with renaissance automata such as Jacques Vaucanson's duck and flute player. Automata, or robots, continue to play an important role in the study of animal and human behavior in what is here called non-interactive, or *classical*, biorobotics. In classical biorobotics, the construction and experimental analysis of robots produces evidence for the study of animal behavior. Early examples of classical biorobotics are thoroughly examined in Cordeschi (2002). More recent examples involve the behavior of lobsters (Grasso et al., 2000), ants (Lambrinos et al., 2000), crickets (Reeve et al., 2005), bats (Bou Mansour et al., 2019), and extinct animals (Long et al., 2006; Long, 2012). Reviews of classical biorobotics are offered in Webb and Consi (2001), Webb (2001), Webb (2002) and Gravish and Lauder (2018).

Recent years have seen the emergence of what appears to be an interactive version of biorobotics. In what is here called *interactive* biorobotics, hypotheses on animal behavior are tested by analyzing how animals interact with robots. Interactive biorobotics has produced scientifically interesting results regarding the behavior of birds^1^, fish^2^, dogs^3^, squirrels^4^, crabs^5^, honeybees^6^, rats^7^, and other animal species^8^. Comprehensive reviews are offered in Krause et al. (2011), Mitri et al. (2013), and Romano et al. (2019b), in which it is also explained why robots, more than non-interacting decoys or computer animations, can be useful for the study of certain aspects of animal behavior.

This article carries out a methodological analysis of interactive biorobotics from the perspective of the philosophy of science. It has two main goals. The first goal is to argue that interactive biorobotics is methodologically different, in some important respects, from classical biorobotics and countless instances of model-based science. The second goal is to reflect on the logic of interactive biorobotics and formulate a provisional non-exhaustive checklist of questions that may be useful to justify and evaluate the validity of interactive biorobotics studies.

The first goal is articulated in two more specific objectives. The first objective is to argue that classical and interactive biorobotics should not be thought of as two variants of the same research strategy. From a methodological point of view, they are, in a sense, on different planets. Interactive biorobotics does not adopt the understanding by building approach, or synthetic method, as defined by many scholars (Cordeschi, 2002; Pfeifer et al., 2008). In classical biorobotics, one builds a robot and analyses its behavior to draw theoretical conclusions about the target living system. The robot serves as a surrogate for reasoning about the target system: it is a surrogate because it replaces the target living system in the experiments. In interactive biorobotics, the role of the robot is totally different. It does not serve as a surrogate for reasoning about the target system, because the target system is there. Rather, it is used to stimulate the target system in ways that are functional to learning something about it. Theoretical conclusions about target-system behavior are not obtained by analyzing the behavior of the robot, but by analyzing the behavior of the target system while it interacts with the robot. Classical and interactive biorobotics substantially differ in the epistemic role assigned to the robot and in the methodological structure. These considerations will be developed in section Interactive Biorobotics is Not the Synthetic Method.

Another specific objective is to argue that the emergence of interactive robotics calls for the revision of our intuitions about what scientific models are for. Interactive biorobots are scientific models (at least, according to two influential definitions of this concept). Philosophers of science and philosophically minded scientists agree that the primary epistemic function of models, in science, is to serve as surrogates to reason about the object they stand for. However, interactive biorobots are not used for surrogative reasoning: they are used to stimulate the system they interact with. Their epistemic role is significantly different from the role typically played by models in physics, chemistry, biology, economics, and other areas of science. Interactive biorobots are therefore epistemically novel: they play a role that is alien to model-based science as we typically conceive it. These considerations will be made in section Interactive Biorobots: Scientific Models or just Robots?

The second goal of this article is to formulate a provisional non-exhaustive checklist of questions that may be useful to evaluate and justify the validity of interactive biorobotics studies. An interactive biorobotics study is valid if the theoretical conclusions are properly supported by the experimental results. It is assumed here that there is no algorithm to decide whether an interactive biorobotics study is valid. However, it is possible to formulate a set of questions that may guide one's analysis of the validity of the study by directing scientists' attention to important aspects of the methodology. The questions formulated in section The Validity of Interactive Biorobotics Studies of this article will mainly concern the structure of the theoretical hypotheses under scrutiny in interactive biorobotics studies and the role of the context. It will be argued that one thing is the long-term research goal that motivates a study, and another thing is the specific theoretical hypothesis that is experimentally corroborated or refuted. All interactive biorobotics studies pursue the long-term goal of contributing to the study of animal behavior. However, a distinction can be made between two classes of studies, which will be called “proximal” and “distal.” In proximal studies, experiments on robot-animal interaction are brought to bear on theoretical hypotheses on robot-animal interaction. In distal studies, experiments on robot-animal interaction are brought to bear on theoretical hypotheses on *animal*-animal interaction. It is important to discern whether a study is proximal or distal, because distal studies involve logical steps that may be particularly hard to justify. The justification of proximal and distal studies also requires reflecting about the relationship between the context in which the experiments were carried out and the context in which the conclusions are expected to hold. The reconstruction of the logic of interactive biorobotics made here, though preliminary, may contribute to justifying the important role that robots, as tools for stimulating living systems, can play in the contemporary life sciences.

## Classic and Interactive Biorobotics: Some Case Studies

This section introduces four cases of biorobotics investigation on animal behavior, which will be used to illustrate and support the theses proposed in this article (e.g., it will be argued that case study 1 exemplifies classic biorobotics, while studies 2, 3, and 4 exemplify forms of interactive biorobotics). This is a representative list only: other studies drawn from the literature cited in the previous section may fit the methodological categories identified in this article—indeed, many additional examples will be made in the next sections. Note also that the purpose of this section is neither to provide a detailed analysis of these studies, nor to comment on their scientific significance. For each study, we will briefly describe the research question, the theoretical hypothesis, the methodology and the results, focusing on the aspects that will turn out to be more relevant to the ensuing discussion, and omitting unnecessary technicalities. The reader is referred to the original articles for more detailed information.

**Case study 1: bat echolocation** (Bou Mansour et al., 2019). In cluttered environments, the sonar system of bats receives a multitude of interfering and overlapping echoes. It is therefore unlikely that bats can localize multiple objects using their sonar system. Bou Mansour et al. (2019) hypothesized that bats use interaural level difference to fly through cluttered environments—i.e., that they compare the loudness of the echoes received by the left and the right ear. Louder left echoes will probably indicate closer obstacles on the left, and vice versa. The authors also hypothesized that bats combine interaural level comparison with acoustic gaze scanning: while flying, bats move their head (therefore, their sonar system) relative to the body axis according to interaural level difference. To test whether interaural level differences combined with acoustic gaze scanning can produce a good obstacle avoidance behavior in cluttered environments, the authors, building on previous simulation studies, implemented two obstacle avoiding strategies in a mobile robot. In one of them, the robot used interaural comparison only, with its gaze rigidly aligned with the body axis (fixed head strategy). In the other one, the robot adjusted its acoustic gaze depending on interaural level difference (acoustic gaze scanning strategy). The two control strategies were tested in two environments, an arena with many obstacles and a corridor similar to the environments typically used to study sonar-based flying in real-life bats. Both environments returned a multitude of interfering echoes to the robot's sensors. In the experimental trials, the fixed head strategy produced a lower number of collisions and a better steering behavior than the acoustic gaze strategy. The authors therefore concluded that, “if the complexity of the environment prevents the bat from inferring the spatial layout of the environment, gaze scanning is disadvantageous” (p. 14). Indeed, “the limited spatial information provided by the interaural differences might not be sufficient to guide the gaze to informative directions. In particular, under these conditions, the cost of not looking where you are going might outweigh the limited benefit of looking around” (p. 14).

**Case study 2: jumping direction in locusts** (Romano et al., 2019a). Does previous exposure to a predator, such as a gecko, affect subsequent jumping escape direction and surveillance orientation in locusts? One hypothesis formulated by the authors is that the escape and surveillance behaviors of locusts are modulated by experience. To test this hypothesis, the authors built a realistic robotic replica of a gecko, which could be made suddenly appear on the test bench, thus simulating a predatory event. In the training phase, different locusts placed in a transparent cage were individually exposed to simulated attacks by the robotic gecko coming from their left or right side, thus producing left-trained and right-trained locusts. A first experimental phase was devoted to the study of jumping escape direction: left-trained and right-trained locusts were exposed to simulated *frontal* attacks by the robotic gecko, and their jumping escape direction was recorded. The experiments showed that previous exposure to the predator affected jumping escape direction: the number of right jumps were higher in left-trained locusts, and vice versa. This was taken by the authors to suggest that real-time escape neural mechanisms are rapidly adaptable. A second experimental phase was devoted to the study of surveillance orientation (locusts use a preferential eye during surveillance). Left-trained and right-trained locusts were kept in a large transparent cage. Five minutes after the introduction of the locust in the cage, the robotic gecko appeared on a nearby bench and performed movements that, according to the literature, can elicit surveillance behavior in locusts. The movements of the locusts were recorded. The results showed no effect of the training phase on surveillance orientation.

**Case study 3: zebrafish shoaling** (Ruberto et al., 2016). Is the behavior of a zebrafish affected by motion and visual appearance of a nearby robotic fish? One hypothesis formulated by the authors is that robots displaying realistic appearance and motion will “attract” real-life zebrafish. To test this hypothesis, they built a robotic fish actuated by a four-degree-of-freedom robotic platform that can produce three-dimensional motion and body oscillations. In each experimental session, the robotic fish was kept in the same pool with an individual zebrafish, separated from the fish by a transparent panel. The behavior of the zebrafish was analyzed in terms of speed and acceleration, distance from the robot, time budgeting along the water column and shoaling tendency. Experimental conditions differed in the motion and visual appearance of the robot. Motion could be three-dimensional, bi-dimensional, or null (static fish). Appearance was manipulated using a realistic replica, an optical transparent model and nothing (only the rod connecting the replica to the actuation mechanism was inserted in the pool). The measurement of zebrafish behavior in the different experimental conditions, along the dimensions summarized above, enabled the authors to conclude that “fish were attracted toward the three-dimensionally moving replica, and this attraction was lost when either its visual appearance or motion was controlled” (p. 11). The target fish was attracted neither by the static realistic replica nor by the moving transparent model. The three-dimensionally moving realistic replica also elicited an increase in speed and acceleration, and a preference for the bottom of the tank. Taken together, these results enabled the authors to conclude that, indeed, realistic appearance and motion determine shoaling behavior in zebrafish.

**Case study 4: gaze following in starlings** (Butler and Fernández-Juricic, 2014). Can starlings recognize the location of conspecific attention? In particular, if one starling gazes at some point around a barrier with a window, will a starling nearby look through the window? The hypothesis formulated by the authors is that starlings can follow the gaze of conspecifics. To test this hypothesis, the authors built two robots, mimicking the shape of a male and a female starling, covered by the skin of a deceased starling, which could rotate the whole body and perform head-down pecking and head-up scanning movements. The experiments were carried out in a three-compartment enclosure: one for the robot, one for a real-life starling, and an empty compartment. The barrier separating the real-life starling and the empty compartment had two windows. For each bird, two experimental conditions were tested: in one, the robot gazed at the empty compartment; in the other one, the robot gazed at the starling. The measured variables were the bird's gaze location and head movement rate (in some bird species, fixation produces an increase in head movement rate). Note that starlings have laterally placed eyes and often perform head movements: determining gaze location as a function of head orientation is a challenging problem, which the authors attempted to solve relying on a number of theoretical assumptions on the visual system of starlings (including assumptions on their visual field configurations and photoreceptor density). Similar assumptions had to be used to decide how to orient the robot so that it gazed at the empty compartment. The results indicated that the probability that the starling would look at the empty compartment through the window was significantly higher when the robot gazed at the empty compartment, compared with when the robot gazed at the starling. Head movement rates were not affected by robot movements except for a peak immediately before reorientation, in some cases. These results were taken to support the initial theoretical hypothesis: “to our knowledge, this is the first report of a non-mammal reorienting its attention geometrically in response to the orientation behavior of conspecifics in a species with laterally placed eyes. This suggests that starlings recognize the location of conspecific attention” (p. 4).

## The Methodological Novelty of Interactive Biorobotics

### Interactive Biorobotics Is Not the Synthetic Method

The case studies described in the previous section are useful to illustrate methodological similarities and differences between *classical* (or non-interactive) and *interactive* biorobotics. In what is here called classical biorobotics, the construction, experimental manipulation, and analysis of the behavior of a stand-alone robot supports theoretical hypotheses concerning animal behavior. In interactive biorobotics, hypotheses on animal behavior are tested by observing how living systems react to the stimuli exerted by robots. Classical and interactive biorobotics share some common methodological features: both research fields

1. involve the construction and experimental manipulation of robots, and
2. aim at testing hypotheses on animal behavior.

**Figure 1:**
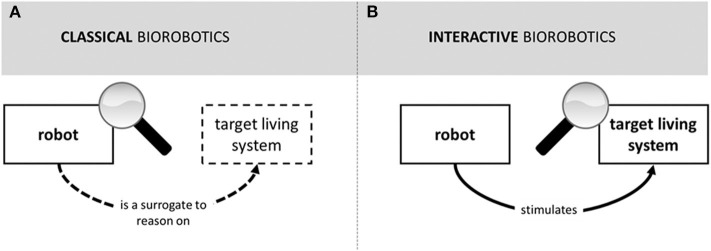
Representation of the basic structure of classical **(A)** and interactive **(B)** biorobotics. In classical biorobotics, the robot is used as a surrogate to reason on the target living system, the robot replaces the target system, the target system is not part of the experimental scenario, and the target of the analysis is the robot. In interactive biorobotics, the robot is used to stimulate the target living system, the robot does not replace the target system, the target system is part of the experimental scenario, and the target of the analysis is the target living system.

The analogy between classical and interactive biorobotics ends here, though. Indeed, the two research fields differ from one another in several respects, as summarized in Table 1 and shown in Figure 1.

**Table 1:** Methodological differences between classical and interactive biorobotics.

	**Classical biorobotics**	**Interactive biorobotics**
(A) What is the role of the robot?	The robot is used as a surrogate to reason about the target system	The robot is used to stimulate the target system
(B) Does the robot replace the target living system?	Yes	No
(C) Is the target living system part of the experimental scenario?	No	Yes
(D) Is the robot the target of experimental analysis?	Yes	No

In classical biorobotics, the robot serves as a surrogate to reason about a target living system (line A in Table 1, left; see also Figure 1A). This means that the robot replaces the target system (B) and that the target system is not part of the experimental scenario (C). The robot is the target of experimental analysis (D): one carries out experiments on the robot in order to draw theoretical conclusions about the behavior of the target system. The use of robots as surrogates for reasoning about biological systems characterizes the so-called understanding by building approach or synthetic methodology based on “using robots *rather than* working with humans or animals” (Pfeifer et al., 2008, 122 emphasis added) in order to study and explain their behavior. The history of the development of the synthetic method has been reconstructed by Cordeschi (2002). The methodological structure of classical biorobotics is analyzed in Webb and Consi (2001), Webb (2001, 2006). See Datteri (2017) and Datteri and Schiaffonati (2019) for a distinction between model-oriented and prediction-oriented classical biorobotic studies.

The logic of interactive biorobotics is very different from the logic of classical biorobotics. Notably, interactive biorobotics does not conform to the synthetic method or understanding by building approach as characterized by Cordeschi (2002) and Pfeifer et al. (2008). From a methodological point of view, it marks a radical point of departure relative to this well-known epistemic use of robotic systems. Indeed, in interactive robotics, the robot is not used as a surrogate to reason about the target system: it is used to stimulate the target system (line A in Table 1, right; see also Figure 1B). This implies that the robot does not replace the target living system (B) and that the target living system is part of the experimental scenario (C). The target of analysis is the target living system and not the robot (D). In classical biorobotics, one analyses the behavior of the robot to draw theoretical conclusions about the target living system. In interactive biorobotics, one analyses the behavior of the target system while it interacts with the robot to draw theoretical conclusions about the target system.

The distinction between classical and interactive biorobotics can be illustrated by reference to the case studies described in section Classic and Interactive Biorobotics: Some Case Studies. The following considerations are summarized in Table 2. In case study 1, the target system is the bat, and the robot is used as a surrogate to reason about its echolocation system (A). The robot replaces a bat (B) and no real-life bat is part of the experimental scenario (C). The robot is the target of experimental analysis (D): the authors carry out experiments on the robot in order to draw theoretical conclusions about the behavior of the bat. This is therefore an example of classical biorobotics (see Table 2, first column).

**Table 2:** Methodological differences between classical and interactive biorobotics, with reference to the four case studies.

	**Case study 1 (Bou Mansour et al., 2019)**	**Case study 2 (Romano et al., 2019a)**	**Case study 3 (Ruberto et al., 2016)**	**Case study 4 (Butler and Fernández-Juricic, 2014)**
A	**The robot is used as a surrogate** to reason about bats	**The robot is used to stimulate** locusts	**The robot is used to stimulate** zebrafish	**The robot is used to stimulate** starlings
B	**Yes**. The target system is the bat, and the robot replaces it	**No**. The target system is the locust, and the robot does not replace it (it replaces a gecko)	**No**. The robot does not replace the focal zebrafish. It replaces a zebrafish that interacts with the focal one	**No**. The robot does not replace the focal starling. It replaces a starling that interacts with the focal one
C	**No**. The experimental scenario does not involve any bat	**Yes**. The experimental scenario involves locusts	**Yes**. The experimental scenario involves zebrafish	**Yes**. The experimental scenario involves starlings
D	**Yes**. The robot is the target of analysis	**No**. The target of analysis is the locust	**No**. The target of analysis is the real-life zebrafish	**No**. The target of analysis is the real-life starling
	**Classical biorobotics**	**Interactive biorobotics**		

Case 2 does not conform to the synthetic method or understanding by building approach, as characterized by Cordeschi (2002) and Pfeifer et al. (2008). The target system is the locust, and the robot is used to stimulate it (A). This implies that the robot does not replace a locust—it indeed replaces a gecko (B)—and that a locust needs to be part of the experimental scenario (C). The target of analysis is the real-life locust and not the robotic gecko (D): it is from the analysis of locust's behavior under robotic stimulation that the authors draw theoretical conclusions about whether previous exposure to a predator affects jumping escape direction and surveillance orientation. The robot, in this case, is not used as a surrogate of locusts to reason about locusts: this is not an example of classical biorobotics. This is a paradigmatic example of interactive biorobotics (see Table 2, second column).

**Figure 2:**
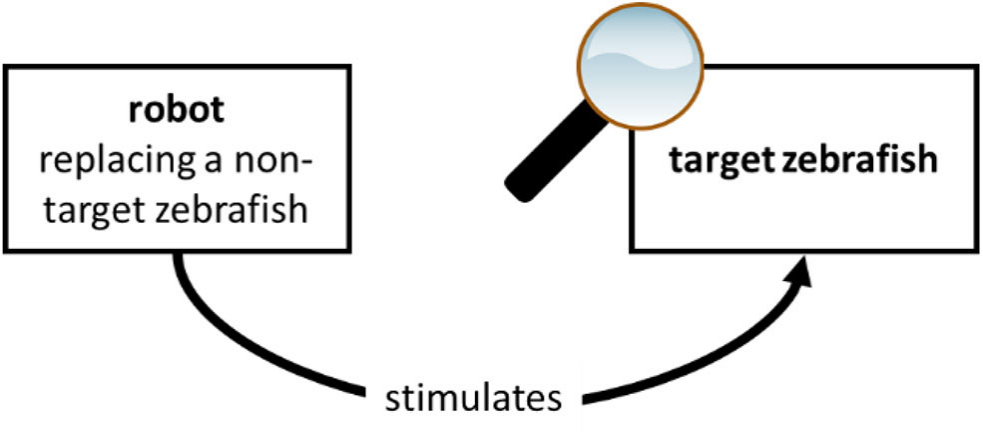
When, in interactive biorobotics, the robot replaces a conspecific, it does not replace the target system.

One may doubt that case studies 3 and 4 qualify as interactive biorobotics studies as defined by the corresponding column in Table 1, for the following reason. Sure enough, in both studies, the robot is used to stimulate the target system, which is a zebrafish and a starling, respectively (A). Sure enough, in both studies, the target system is part of the experimental scenario (C) and the robot is not the target of analysis (D): in the experiments, the authors analyse the behavior of real-life zebrafish and starlings under the stimulations exerted by the robot. However, one may be tempted to answer “yes” to question B, thus excluding cases 2 and 3 from the interactive biorobotics category. Indeed, in the two studies, the robot replaces a zebrafish and a starling, respectively, i.e., a *conspecific* of the target system [while, in case study 2 and in other prey-predator studies such as Polverino et al. (2019), and Romano et al. (2020a) the robot replaces an individual of a different species]. For this reason, one may be tempted to believe that the robot replaces the target system: the target system is the zebrafish and the starling, respectively, and the robot replaces a zebrafish and a starling, respectively. One should resist that temptation, however, and answer “no” to question B. It is true that, in study 3, the robot replaces a zebrafish, and that the target system is a zebrafish. But the target zebrafish is *not* the zebrafish replaced by the robot. The goal of the study is not to learn anything about *how the replaced zebrafish would behave* in its portion of the pool. Neither could it be used for that purpose, because the behavior of the robot is pre-programmed, thus poorly informative of its autonomous behavior. Thus, it is true that the robot replaces a zebrafish, but it is false that it replaces the target or focal zebrafish, as schematised in Figure 2. For this reason, question B must be answered in the negative. Similar considerations can be made concerning study 4. They both conform to the interactive biorobotics approach, as defined in Table 1; see also Table 2, third and fourth column.

To sum up. In interactive biorobotics, one builds robotic systems to understand animal behavior. Nevertheless, interactive biorobotics does not exemplify the understanding by building approach or synthetic method as commonly defined and applied so far. In the next section we will argue that interactive biorobotics calls for a change in our views about what scientific models are for.

### Interactive Biorobots: Scientific Models or Just Robots?

Classical and interactive biorobotics involve the construction and analysis of what may be called robotic *models* of living systems. Robotic models of bats, lobsters, ants, and portions of the human nervous system are built in classical biorobotics. Robotic models of geckos, zebrafish, starlings, interact with living systems in interactive biorobotics. Use of concrete systems *qua* scientific models of other concrete systems is pervasive in science. However, here it will be suggested that interactive biorobots should be regarded as models of a novel epistemic variety: their epistemic role—i.e., the role they play in the acquisition of new knowledge about the world—is significantly different from the role played by models in other model-based areas of scientific research, classical biorobotics included. To reach this conclusion, we will have to argue

- that interactive and classical biorobots can be called scientific models of living systems;
- that the epistemic role typically played by models in science, classical biorobotics included, is to serve as surrogates for reasoning about the system they stand for; and
- that interactive biorobots are not used for surrogative reasoning (this point was made in section Interactive Biorobotics Is Not the Synthetic Method).

Taken together, A, B, and C lead to the conclusion that interactive biorobots do not play the epistemic role typically played by models in science.

**Figure 3:**
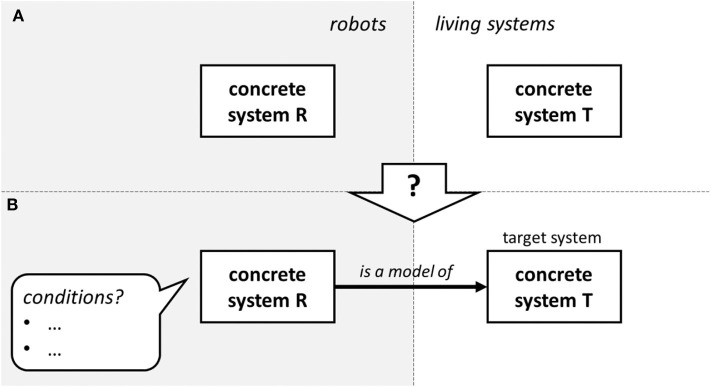
Prima facie, what we call a (robotic) model R is just a concrete system. **(A)** Under what conditions **(B)** can it be regarded as a scientific model of another concrete (living) system T?

Let us start from point A: interactive and classical biorobots can be regarded as scientific models of living systems. What is a scientific model? In scientific research, concrete systems are often regarded as models of other systems. A double spiral made of plastic can be regarded as a model of the DNA helix. A gigantic concrete platform filled with water can be regarded as a model of San Francisco Bay (Weisberg, 2015). A hydraulic mechanism (the MONIAC, or Phillips Hydraulic Computer, built by William Phillips, 1949) can be regarded as a model of the British economy. The robots used in case study 1 and 2 can be regarded as models of bats and geckos, respectively. *Prima facie*, these systems are just concrete systems (Figure 3A, left). Under what conditions can they be regarded as models of other systems (Figure 3B, left)? By identifying necessary and sufficient conditions for a concrete system to be a model of another concrete system, one defines the concept of model. Indeed, a definition of the concept of model can be expressed as a statement of the form “R is a model of target system T if and only if …,” where the dots are replaced by a list of conditions. Various families of definitions have been formulated, chiefly including the so-called similarity-based and inferential ones (Frigg and Nguyen, 2017).

According to one possible intuition, *resemblance* to the modeled object is what makes a concrete object a model of it. This intuition has been developed in the so-called *similarity* conception of model. According to the similarity conception (Giere, 1990), R is a model of T if and only if R is similar to T in scientifically relevant aspects. This position has some intuitive plausibility: indeed, scientists who want to build concrete models typically try to build systems that resemble the target system in scientifically relevant aspects (in biorobotics, they typically try to build *biomimetic* robots). However, a more careful reflection reveals that this cannot be the whole story. Leaving aside the problem of defining similarity, my car may be similar to your car, but something more than this fact would be needed to justify calling my car a model of your car (Toon, 2012). Moreover, similarity is a symmetrical relation: if R is similar to T, T is similar to R. But many of us would deny that, if a robot is a model of a bat, then one should be justified in regarding a bat as a scientific model of that robot. It is reasonable to demand that a definition of model be asymmetrical. For these reasons, many scholars believe that similarity, *alone*, cannot define what turns a concrete object into a model^9^.

Another conception of model, called *inferential*, was proposed by Suárez (2004). According to the inferential conception, R is a model of T if two conditions are satisfied:

1. an agent A stipulates that R represents T, and
2. R allows A to draw specific inferences regarding T.

According to the first condition, the robot in case study 1 is not a model of a bat unless some agent takes it to represent a bat. This condition captures the intuition that nothing is a model *per se*, and that R would not be a model if nobody regarded it as such. According to the second condition, the robotic bat is a model of a bat if it enables the agent to draw specific inferences regarding bats, or, in more familiar terms, if it enables them to carry out forms of *surrogative reasoning* about bats (Swoyer, 1991). Note that Condition 2 does not require that the conclusions of these inferences be true: there are “good” and “bad” models. If a robot enables one to draw false conclusions about bats, it will be a *bad* model of them, but still a *model*. Note also that the inferential conception is asymmetrical. R being a model of T does not imply that T is a model of R, because there is an agent A who stipulates that R is a model of T without necessarily stipulating the converse (condition 1).

Can the robots used in classical and interactive biorobotics be regarded as models of living systems? Let us try to address this question by determining whether the robotic bat involved in case study 1 (an example of classical biorobot) and the robotic gecko involved in case study 2 (an example of interactive biorobot) satisfy the two definitions above. It is reasonable to believe that they both satisfy the similarity conception: the robotic bat resembles a bat in the control mechanism, and the robotic gecko resembles a gecko in the appearance (see Table 3). Indeed, classical and interactive bioroboticists attempt to build biomimetic robots (see Datteri, 2017 for a discussion on biomimicry in classical biorobotics). They satisfy the inferential definition too. Both robots are taken by the authors of the two studies to represent a living system, a bat and a gecko, respectively (condition 1), and one may use the robotic bat and the robotic gecko to infer conclusions about bats and geckos (condition 2). If these conclusions were wrong, one could claim that they are bad models—but they would be models, nonetheless. It is reasonable to believe that these considerations can be generalized to many other cases of classical and interactive biorobotics, thus leading us to conclude that classical and interactive biorobots are models.

**Table 3:** Classical and interactive biorobotics are models, according to the similarity and inferential conceptions.

	**Similarity conception**	**Inferential conception**	
		**Condition 1**	**Condition 2**
Classical biorobotics (Bou Mansour et al., 2019)	**Yes**. The robotic bat resembles a bat in the control mechanism	**Yes**. The robotic bat is taken by the authors to represent a bat	**Yes**. The robotic bat can be used to draw inferences about bats
Interactive biorobotics (Romano et al., 2019a)	**Yes**. The robotic gecko resembles a gecko in the external appearance	**Yes**. The robotic gecko is taken by the authors to represent a gecko	**Yes**. The robotic gecko can be used to draw inferences about geckos

So far, we have taken the first step (A) of the argument outlined at the beginning of this section: we have argued that interactive and classical biorobots can be regarded as scientific models of living systems. Let us now turn to point B. How are models typically used in science? There are good reasons to claim that the epistemic role typically played by models in science, classical biorobotics included, is to serve as surrogates for reasoning about the system they stand for. In biology, models of the DNA molecule are used to reason about the characteristics of the DNA molecule. The concrete model of the San Francisco Bay described in Weisberg (2015) is used to reason about water flow in the San Francisco Bay. The MONIAC was used to reason about the dynamics of British economy. This is true of classical biorobotics too, as argued in section Interactive Biorobotics Is Not the Synthetic Method: the robotic bat involved in case study 1, for example, was used to reason about the sensory-motor mechanisms of bats. Most scholars, including Suárez (2004) and Swoyer (1991), agree that surrogative reasoning is the basic function of scientific models. In a systematic review of the debate Frigg and Nguyen (2017, p. 55) assume that “models represent their targets in a way that allows us to generate hypotheses about them,” and claim that any acceptable definition of model will have to account for “how reasoning conducted on models can yield claims about their target systems” (Frigg and Nguyen, 2017, p. 51).

These are powerful reasons to believe that the epistemic role typically played by models in science, classical biorobotics included, is to serve as surrogates for reasoning about the system they stand for (point B in the argument outlined above). The thesis expressed in point C was defended in section Interactive Biorobotics Is Not the Synthetic Method: interactive biorobots are not used as surrogates to reason about the system they stand for. They are used to stimulate the system they interact with.

Taken together, points A, B, and C enable us to conclude that interactive robots are models of a novel epistemic variety. They *are* models: they are not just mere objects, deprived of any modeling function. Romano et al. (2019a) built a robotic model of a gecko, Ruberto et al. (2016) built a robotic model of a zebrafish, and Butler and Fernández-Juricic (2014) built a robotic model of a starling. These systems are scientific models according to influential definitions of the concept. They also contribute to scientific research: they have an epistemic role. However, their role is significantly different from the role typically played by models in physics, chemistry, biology, economics, and other areas of science. It is in this sense that interactive biorobots are epistemically novel: they play an epistemic role that is alien to model-based science, as we typically conceive it. Whether the emergence of interactive biorobotics challenges our conception of a what a scientific model *is*, is a question deferred to future studies. Sure enough, however, it urges us to change our intuitions about what scientific models *are for*.

## The Validity of Interactive Biorobotics Studies

### What Do We Learn in Interactive Biorobotics? Proximate and Distal Studies

So far, it has been argued that interactive robots can be regarded as scientific models and used to acquire new knowledge about animal behavior in a way that departs from the synthetic method and classical biorobotics. The structure of the synthetic method has been extensively discussed by many scholars (Webb, 2001; Cordeschi, 2002; Datteri, 2017). These authors reflect on whether the results of *classical* biorobotics experiments can be validly used to support or refute theoretical hypotheses about animal behavior. Can *interactive* biorobotics experiments be validly used to support or refute theoretical hypotheses about animal behavior? How to evaluate the validity of interactive biorobotics studies? Few methodological analyses of this methodology have been carried out so far (Mitri et al., 2013; Datteri, 2020). By reconstructing the logic of interactive biorobotics, this section will offer a provisional and non-exhaustive checklist of questions that may guide validity evaluation.

This reconstruction starts from a distinction between the research goals and the theoretical hypotheses that are corroborated or rejected in a study, and from a distinction between two broad classes of interactive biorobotics studies, called *proximal* and *distal*, which differ from one another in the structure of the theoretical hypothesis under investigation. In the next section, it will be argued that distal studies distinctively involve logical steps that can be particularly hard to justify.

A first, basic distinction is between the long-term research goals motivating a study and the specific theoretical hypothesis that is ultimately corroborated or rejected^10^. A study may be motivated by the long-term research ambition to understand the dynamics of zebrafish schooling, and test a more specific theoretical hypothesis on the impact of the appearance and type of motion of a robot on particular aspects of the behavior of a zebrafish. Indeed, research goals often consist in long-term research ambitions and are more general than the specific hypothesis under scrutiny. Properly distinguishing between the long-term goals and the theoretical hypothesis tested in a study is important for at least two reasons. At least in one sense of the term, validity obtains if the experimental results are properly brought to bear on the theoretical hypothesis, regardless of the broader research goal. More interestingly, it will be argued that, although all interactive biorobotics studies share the long-term ambition to understand the dynamics of *animal*-animal interaction, some studies test theoretical hypotheses on *robot*-animal interaction. This leads us to the first and most basic question of the checklist.

(Q1) What is the theoretical hypothesis under investigation in the study, as distinct from the (possibly more general) research goal?

Under a certain level of approximation, the theoretical hypotheses under scrutiny in interactive biorobotics studies share a common structure. They all state that the behavior of the target animal will be such and such whenever some triggering conditions obtain. For example, one of the hypotheses under scrutiny in case study 3 (Ruberto et al., 2016) states that zebrafish will be attracted to a robot when it displays a realistic appearance and 3d motion. The hypothesis corroborated in case study 4 (Butler and Fernández-Juricic, 2014) states that one starling will tend to direct its attention toward a particular point when a starling nearby directs its attention to the same point. Let us assume that the behavior of the target system can be described by parameters that may take values. For example, in Ruberto et al. (2016), the behavior of the target zebrafish is described in terms of speed and acceleration, distance from the robot, time budgeting along the water column, and shoaling tendency—all of them being parameters that can take numerical values. The behavior of the focal starling, in Butler and Fernández-Juricic (2014), is described in terms of the probability that it will gaze at a particular point and of head movement rate. Thus, the theoretical hypotheses that are corroborated or rejected in interactive biorobotics take the form of law-like generalizations of the form “whenever some triggering conditions obtain, the parameters describing the target behavior will assume such and such values,” or, schematically,

$$\begin{array}{l} \text{triggering conditions }\to {\text{P}}_{\text{T}}=<pt_{1}=v_{\text{p}},\ldots ,pt_{\text{n}}=v_{\text{q}}> \end{array}$$

where P_T_ (“P” for parameter and “T” for target system) is the set of parameters pt_1_, …, pt_n_ defining the relevant aspects of the target system behavior. The set P_T_ includes parameters representing speed and acceleration, distance from the robot, time budgeting along the water column, and shoaling tendency in Ruberto et al. (2016), and parameters representing gaze probability and head movements rate in Butler and Fernández-Juricic (2014).

**Figure 4:**
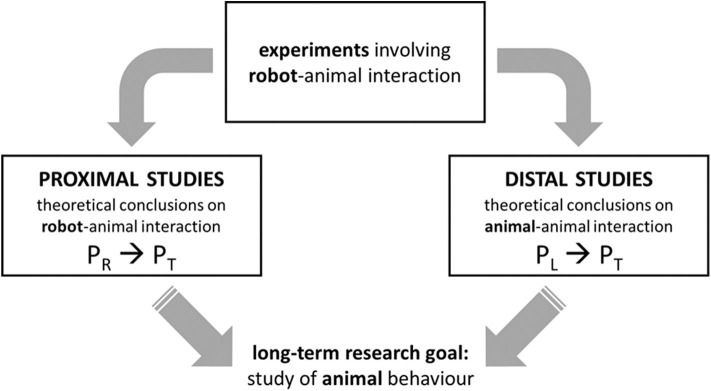
Proximal and distal studies.

At least two families of studies can be identified, differing from one another in the nature of the triggering conditions (see Figure 4). In what we will here call *proximal* studies, the triggering parameters describe a robot and the hypothesis under scrutiny concerns the dynamics of robot-animal interaction. The study published in Ruberto et al. (2016) is an example of a proximal study. The long-term research goal was to understand the dynamics of animal-animal interaction in zebrafish at large, but the authors ultimately corroborated a hypothesis on robot-animal interaction stating that robots having a certain appearance (realistic) and displaying a particular motion (3d) attract zebrafish. The hypothesis establishes a law-like regularity between the value of certain parameters describing the robot, and the value of certain parameters describing the behavior of the target system, as in the following schema:

$$\begin{array}{l} P_{\text{R}}\;\to \;P_{\text{T}} \end{array}$$

where P_R_ (“P” for parameter and “R” for robot) is the set of parameters pr_1_, …, pr_n_ defining the relevant aspects of the robot (e.g., appearance and motion). Other proximal studies are, for example, (Abaid et al., 2012; Polverino and Porfiri, 2013b; Jolly et al., 2016; Griparić et al., 2017; Bierbach et al., 2018). All these studies had the long-term goal of understanding the dynamics of animal-animal interaction but tested a more specific hypothesis on how animals react to a robot. Evaluating validity in proximal studies involves evaluating whether experiments on robot-animal interaction properly support a hypothesis concerning robot-animal interaction. As we shall discuss, there is a relatively short “distance” between experimental results and theoretical conclusions in proximal studies.

In what are here called *distal* studies, experiments on *robot*-animal interaction are brought to bear on hypotheses concerning *animal*-animal interaction. An example is case study 4 (Butler and Fernández-Juricic, 2014). As reported in section Classic and Interactive Biorobotics: Some Case Studies, the authors observed the interaction between real-life starlings and starling-like robots. The experimental results, chiefly concerning robot-animal interaction, were taken to support a theoretical hypothesis on animal-animal interaction: “to our knowledge, this is the first report of a non-mammal reorienting its attention geometrically in response to the orientation behavior *of conspecifics* in a species with laterally placed eyes. *This suggests that starlings recognize the location of conspecific attention*” (p. 4, emphasis added). The theoretical hypothesis is a law-like regularity between parameters describing the behavior *of one living system* (a conspecific) and parameters describing the behavior of *another living system* (the target animal), as in the following schema:

$$\begin{array}{l} P_{\text{L}}\;\to \;P_{\text{T}} \end{array}$$

where P_L_ is the set of parameters describing the living system which the robot stands for (i.e., another starling). Other distal interactive biorobotics studies are (Michelsen et al., 1992; Reaney, 2009; Margerie et al., 2011; Polverino and Porfiri, 2013a; Romano et al., 2017).

In short, in proximal studies, experiments on robot-animal interaction are brought to bear on hypotheses on robot-animal interaction. A relatively short distance must be traveled from the experimental results to the conclusions. In distal studies, experiments on robot-animal interaction are brought to bear on hypotheses on animal-animal interaction: the distance between the result of the experiment and what is asserted by the theoretical conclusion is somehow larger. Distal studies involve a logical step that is missing in proximal studies and needs justification. Thus, to evaluate whether an interactive biorobotics study is valid or not, it is important to realize whether it is proximal or distal.

(Q2) Is the study a proximal or a distal one?

### Neutralizing the Context

**Figure 5:**
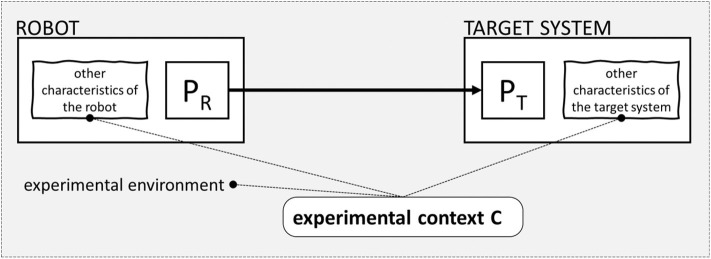
The structure of interactive biorobotics experiments.

To validly bring experimental results to bear on proximal or distal theoretical hypotheses, one must properly reflect on the non-manipulated characteristics of the experimental context. In interactive biorobotics experiments, one selectively manipulates some features of a robot in a suitable experimental environment and observes how the target system reacts. More schematically, some P_R_ parameters of the robot are manipulated, and the behavior of the target system, described by P_T_, is measured, leading to results of the form P_R_ → P_T_ (see Figure 5). In case study 3, Ruberto et al. (2016) manipulated the appearance and type of motion of a robotic zebrafish and measured speed and acceleration, distance from the robot, time budgeting along the water column and the shoaling tendency of the target zebrafish, concluding that certain values of P_R_ determine certain values of P_T_ in that experimental context. Butler and Fernández-Juricic (2014) manipulated the gaze location of the robots and observed gaze location of the focal starling, concluding that the former has some effect on the latter. These manipulations led to experimental results of the form P_R_ → P_T_.

Note that these experiments are context-dependent and enable one, at best, to expect that P_R_ → P_T_ will hold within the background conditions in which the experiments have been carried out. Let us use label C to denote the set of those background conditions, which may be related to the characteristics of the experimental environment, of the robot and of the target system (see Figure 5). Some background characteristics of the environment (e.g., the temperature of water in an experiment on fish behavior) may have had a non-negligible effect on P_T_. Some background characteristics of the robot, for example, the dimension of the rod connecting the replica to the external actuator in Ruberto et al. (2016), may have had an impact on P_T_ too, and some background characteristics of the target system, for example its physiological state, may have had some significant effect on its behavior.

Proximal and distal hypotheses are seemingly context-free, meaning that they apparently make no reference to the context in which they are assumed to hold. Literally, “starlings recognize the location of conspecific attention” is a strict generalization affirming that all starlings will recognize the location of a starling nearby, regardless of the context. Various strategies may be adopted to safely generalize context-dependent experimental results. Contextual influences may be detected by selectively altering features of the context in control experiments and may be neutralized by carrying out additional measurements or theoretical reflections. For example, in Polverino et al. (2013), water perturbation introduced by the rod connecting the robot to the replica was neutralized by not monitoring the behavior of the focal fish around the rod. These experimental strategies and theoretical considerations will not suffice to spot and exclude all the potential sources of disturbance, and the experimental results obtained within a single study will be unavoidably relative to a residual set of non-investigated contextual factors. Some extra assumptions will therefore be needed to validly bring context-dependent results to bear on seemingly context-free theoretical conclusions.

Part of the problem, however, concerns the very meaning of theoretical hypotheses such as “starlings recognize the location of conspecific attention.” The meaning of scientific laws, and the possibility of asserting strict and context-free generalizations in apparently exception-ridden scientific domains such as life sciences, are subjects of long-standing debates in the philosophy of science (Carroll, 1994). According to some scholars (including Woodward, 2000), when a scientist formulates a literally strict and context-free law-like generalization, they have some idea on the context in which that generalization is expected to hold. Let us call it the privileged context C^*^ of the generalization. For example, when the authors of the starling study conclude that “starlings recognize the location of conspecific attention,” they are implicitly referring to a privileged context in which this regularity is expected to hold—which does not encompass, for example, conditions in which the temperature is below −20°C. A legitimate aspiration to generality often makes scientists quite silent on the privileged context in which the theoretical conclusions are expected to hold, so that, in many cases, experimental results obtained in very peculiar conditions are brought to bear on literally universal generalizations. A more careful identification of the privileged context, however, may contribute to shortening the distance between the experimental results and the theoretical conclusion, and to improve the methodological strength of the study. Another question that may guide reflection on the validity of a study might therefore be the following.

(Q3) What is the privileged context in which the theoretical conclusion is expected to hold?

### Justification of Distal Hypotheses

**Figure 6:**
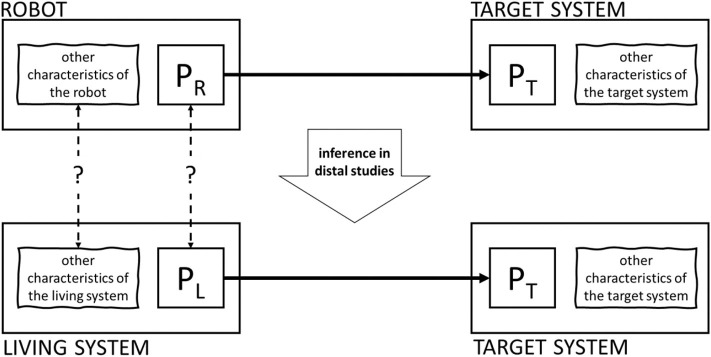
A representation of the logic of distal studies.

An extra reflection on the background characteristics of the robot is needed to justify distal theoretical hypotheses. In distal studies, experiments on robot-animal interaction (showing that P_R_ → P_T_ holds in the experimental context C) are brought to bear on theoretical hypotheses concerning animal-animal interaction of the form P_L_ → P_T_ (in the privileged context C^*^). There is some distance to be covered from finding that some features *of the robot* produce some effects on the target system to concluding that the same features *displayed by an animal* will have the same effects on the target system. The same features, when displayed by an animal, may have different effects because the robot and the animal differ from each other in some background, non-manipulated characteristics. To justify this logical step, which is unique to distal studies, one must reflect on the relationship between the robot and the living system “replaced” by the robot. More specifically, as represented in Figure 6, one must reflect on the relationship between P_R_ and P_L_, on the one hand, and between the background characteristics of the robot R and the characteristics of the living system L replaced by the robot, on the other hand (Datteri, 2020).

First, one must reflect on the relationship between the manipulated parameters of the robot and the corresponding features of system replaced by the robot (P_R_ and P_L_, respectively). Discovering that certain features of the robot have some effects on the target system enables one to safely infer that the same features, when displayed by an animal, will have the same effects on the target system only provided that the two systems really display *the same features*. This consists, in some cases, in ensuring that the robot realistically reproduces the features that are supposed to be displayed by the animal. The reaction of a locust to a robotic gecko in case study 2 (Romano et al., 2019a) can be used to infer that locusts will display the same reaction to geckos only provided that the robot realistically mimics geckos in the relevant aspect. This is akin to the signal fidelity problem that is discussed in Powell and Rosenthal (2017) in connection with computer animations and decoys.

This logical step may be particularly difficult to justify if P_R_ and P_L_ do not describe visual characteristics only. For example, Butler and Fernández-Juricic (2014) “manipulated the visual attention of the robot and measured changes in head and body orientation of a live bird” (p. 2). By “visual attention” they mean gaze location. They discovered that, when the robot was gazing at one point, say A, the target system tended to gaze at A too. This discovery was used to infer that starlings recognize the location of conspecific attention, implying that, if one real-life starling gazes at A, a starling nearby will tend to gaze at A too. The authors jumped from P_R_ → P_T_ experimental results to a P_L_ → P_T_ theoretical conclusion. This jump is justified only if the authors succeed in reproducing the gazing behavior of starlings. One must ensure that the robot produces the same behavior that starlings produce when gazing at A, otherwise it is not clear how the experimental results can be used to reveal gaze following phenomena among real-life starlings. Justifying this logical jump can be challenging. Realistic reproduction of gazing behavior can be relatively unproblematic in humanoid robots, but, as pointed out in section Classic and Interactive Biorobotics: Some Case Studies, starlings have laterally placed eyes: a network of background theories and assumptions concerning the visual field configurations and the retinal distribution of photoreceptors in the two eyes are needed to infer gaze location from behavior in real-life starlings, and to reproduce behavior equivalent to “gazing at A” in a robot. Justifying the logical jump from manipulation of P_R_ to manipulation of P_L_ requires one to justify the solidity of these theories and assumptions. These considerations lead to the following methodological question.

(Q4) In a distal study, what relationship holds between the manipulated characteristics of the robot (described by P_R_) and the features that are claimed to influence the behavior of the target system in animal-animal interaction (described by P_L_)?

Second, to bring experimental results on robot-animal interaction to bear on results concerning animal-animal interaction in distal studies, one must reflect on the relationship between the background (non-manipulated) characteristics of the robot R and the background characteristics of the system L replaced by the robot. Butler and Fernández-Juricic (2014) concluded that starlings recognize the location of conspecific attention. Assuming that the robot accurately reproduced starlings' gazing behavior, other non-manipulated characteristics of the robot, which have no counterpart in real-life starlings, may have produced peculiar reactions in the target system. The authors gave the robots a realistic appearance by covering them with the skin of a deceased bird, but other non-modeled characteristics of the animal, possibly concerning other sensory (olfactory or auditory) cues, might have made the difference. The reaction of the target system to the robot might therefore have been different from the reaction that the target system would have produced to a real-life starling gazing at the same location. This consideration leads us to the following question.

(Q5) In a distal study, what relationship holds between the non-manipulated background characteristics of the robot and the characteristics of the animal which are not mentioned in the theoretical hypothesis?

## Conclusion: A Methodological Checklist for Interactive Biorobotics

This article had two goals. The first one was to emphasize the methodological novelty of interactive biorobotics relative to classical biorobotics and countless instances of model-based science. It was shown that, in interactive biorobotics, the robot is not used as a surrogate for reasoning about the target system. This is why interactive and classical biorobotics are strikingly different from one another from a methodological point of view. Some reasons to believe that interactive biorobots are scientific models of a novel epistemic variety were offered.

The second goal was to reflect on the logic of interactive biorobotics and identify some methodological questions that may be used as a guideline to evaluate the validity of interactive biorobotics studies. They are:

(Q1) What is the theoretical hypothesis under investigation in the study, as distinct from the (possibly more general) research goal?
(Q2) Is the study a proximal or a distal one?
(Q3) What is the privileged context in which the theoretical conclusion is expected to hold?
(Q4) In a distal study, what relationship holds between the manipulated characteristics of the robot (described by P_R_) and the features which are claimed to influence the behavior of the target system in animal-animal interaction (described by P_L_)?
(Q5) In a distal study, what relationship holds between the non-manipulated background characteristics of the robot and the characteristics of the animal which are not mentioned in the theoretical hypothesis?

Question Q1 establishes a distinction between the long-term research goals of a study and the theoretical hypothesis which is supported or refuted. Making this distinction, as basic as it may seem, is important for two reasons. First, validity, as conceived here, concerns the relationship between the experimental results and the theoretical hypothesis (regardless of the research goal). Second, even though all interactive biorobotics studies pursue the long-term goal of understanding the dynamics of animal-animal interaction, some of them test hypotheses on robot-animal interaction. This leads us to the second question. The theoretical hypotheses tested in interactive biorobotics concern the behavior that the target system would produce while interacting with another system, which can be a robot or another animal. Establishing whether the study is a proximal or a distal one (Q2) is important for some reasons. The justification of both proximal and distal hypotheses must involve a careful reflection on the relationship between the context in which the experiment has been carried out and the context in which the theoretical hypothesis is supposed to hold, here called the privileged context. Clarifying the privileged context and avoiding hyper-ambitious generalizations may be helpful in justifying this relationship (question Q3). However, the justification of distal hypotheses requires one to justify the relationship between the robot that was used in the experiment and the animal which it stands for, in terms of the relationship between parameters P_R_ and P_L_ (question Q4) and between the background characteristics of R and L (question Q5).

The goals of this article did not include an evaluation of the validity of particular interactive biorobotics studies. It was not claimed here that the studies carried out so far, and cited in the introduction, need further methodological justification. It was only claimed that the questions identified here can be used to guide a reflection on the validity of past or future studies. More importantly, they may be refined and complemented with further methodological questions. The broadest ambition of this article was to stimulate a debate on the logic of interactive biorobotics from the point of view of philosophy of science, which may ultimately produce norms for carrying out methodologically “good” studies and produce solid reasons to believe that interactive biorobots can significantly contribute to the study of animal behavior.

## Data Availability Statement

The original contributions presented in the study are included in the article/supplementary material, further inquiries can be directed to the corresponding author.

## Author Contributions

The author confirms being the sole contributor of this work and has approved it for publication.

## Conflict of Interest

The author declares that the research was conducted in the absence of any commercial or financial relationships that could be construed as a potential conflict of interest.

## Footnotes

^1^See (Patricelli et al., 2002, 2006; Göth and Evans, 2004; Margerie et al., 2011; Anderson et al., 2013; Butler and Fernández-Juricic, 2014; Jolly et al., 2016).

^2^See (Faria et al., 2010; Gribovskiy et al., 2010; Abaid et al., 2012, 2013; Kopman et al., 2012; Marras and Porfiri, 2012; Polverino et al., 2012, 2013, 2019; Butail et al., 2013, 2014a,b; Cianca et al., 2013; Polverino and Porfiri, 2013a; Spinello et al., 2013; Landgraf et al., 2014, 2016; Phamduy et al., 2014; Worm et al., 2014; Bartolini et al., 2016; Bonnet et al., 2016; Donati et al., 2016; Ruberto et al., 2016; Romano et al., 2017, 2020b; Bierbach et al., 2018, 2020).

^3^See (Kubinyi et al., 2004; Gergely et al., 2013, 2015).

^4^See (Rundus et al., 2007; Partan et al., 2009).

^5^See (Reaney et al., 2008; Reaney, 2009).

^6^See (Michelsen et al., 1992; Landgraf et al., 2008, 2012; Griparić et al., 2017).

^7^See (Shi et al., 2013, 2015).

^8^See (Martins et al., 2005; Fernández-Juricic et al., 2006; Halloy et al., 2007; Ord and Stamps, 2008; Taylor et al., 2008; Partan et al., 2011; Romano et al., 2019a, 2020a).

^9^For additional arguments against the similarity conception, see (Frigg, 2006); see (Bueno and French, 2011) for an analysis of the so-called structural conception, which analyses similarity in terms of structural identity.

^10^For the sake of simplicity, it will be assumed here that just one theoretical hypothesis is tested in every single study. This is clearly false, as many theoretical hypotheses may be tested in the same study. This simplification, however, does not undermine the arguments proposed here.

## References

[B1] Abaid N. Bartolini T. Macrì S. Porfiri M. (2012). Zebrafish responds differentially to a robotic fish of varying aspect ratio, tail beat frequency, noise, and color. Behav. Brain Res. 233, 545–553. 10.1016/j.bbr.2012.05.047 22677270

[B2] Abaid N. Marras S. Fitzgibbons C. Porfiri M. (2013). Modulation of risk-taking behaviour in golden shiners (Notemigonus crysoleucas) using robotic fish. Behav. Processes 100, 9–12. 10.1016/j.beproc.2013.07.010 23876393

[B3] Anderson R. C. DuBois A. L. Piech D. K. Searcy W. A. Nowicki S. (2013). Male response to an aggressive visual signal, the wing wave display, in swamp sparrows. Behav. Ecol. Sociobiol. 67, 593–600. 10.1007/s00265-013-1478-9

[B4] Bartolini T. Mwaffo V. Showler A. Macrì S. Butail S. Porfiri M. (2016). Zebrafish response to 3D printed shoals of conspecifics: the effect of body size. Bioinspir. Biomimet. 11:026003. 10.1088/1748-3190/11/2/026003 26891476

[B5] Bierbach D. Landgraf T. Romanczuk P. Lukas J. Nguyen H. Wolf M.. (2018). Using a robotic fish to investigate individual differences in social responsiveness in the guppy. R. Soc. Open Sci. 5:181026. 10.1098/rsos.181026 30225087 PMC6124066

[B6] Bierbach D. Mönck H. J. Lukas J. Habedank M. Romanczuk P. Landgraf T.. (2020). Guppies prefer to follow large (robot) leaders irrespective of own size. Front. Bioeng. Biotechnol. 8:441. 10.3389/fbioe.2020.00441 32500065 PMC7243707

[B7] Bonnet F. Kato Y. Mondada F. (2016). Infiltrating the zebrafish swarm: design, implementation and experimental tests of a miniature robotic fish lure for fish-robot interaction studies. Artif Life Robot. 21, 18–25. 10.1007/s10015-016-0291-8

[B8] Bou Mansour C. Koreman E. Steckel J. Peremans H. Vanderelst D. (2019). Avoidance of non-localizable obstacles in echolocating bats: a robotic model. PLoS Comput. Biol. 15:e1007550. 10.1371/journal.pcbi.1007550 31856162 PMC6941896

[B9] Bueno O. French S. (2011). How theories represent. Br. J. Philos. Sci. 62, 857–894. 10.1093/bjps/axr010

[B10] Butail S. Bartolini T. Porfiri M. (2013). Collective response of zebrafish shoals to a free-swimming robotic fish. PLoS ONE 8:e76123. 10.1371/journal.pone.0076123 24146825 PMC3797741

[B11] Butail S. Ladu F. Spinello D. Porfiri M. (2014a). Information flow in animal-robot interactions. Entropy 16, 1315–1330. 10.3390/e16031315

[B12] Butail S. Polverino G. Phamduy P. Del Sette F. Porfiri M. (2014b). Influence of robotic shoal size, configuration, and activity on zebrafish behavior in a free-swimming environment. Behav. Brain Res. 275, 269–280. 10.1016/j.bbr.2014.09.015 25239605

[B13] Butler S. R. Fernández-Juricic E. (2014). European starlings recognize the location of robotic conspecific attention. Biol. Lett. 10:20140665. 10.1098/rsbl.2014.0665 25319821 PMC4272211

[B14] Carroll J. W. (1994). Laws of Nature. Cambridge, UK: Cambridge University Press.

[B15] Cianca V. Bartolini T. Porfiri M. Macrì S. (2013). A robotics-based behavioral paradigm to measure anxiety-related responses in zebrafish. PLoS ONE 8:e69661. 10.1371/journal.pone.0069661 23922773 PMC3726767

[B16] Cordeschi R. (2002). The Discovery of the Artificial. Behav Mind Machines Before and Beyond Cybernetics. Dordrecht: Springer.

[B17] Datteri E. (2017). Biorobotics, in Springer Handbook of Model-Based Science, eds Magnani L. Bertolotti T. (Cham: Springer International Publishing, 817–37.

[B18] Datteri E. (2020). Interactive biorobotics. Synthese. 10.1007/s11229-020-02533-2. [Epub ahead of print]. PMC7360728 32733858

[B19] Datteri E. Schiaffonati V. (2019). Robotic simulations, simulations of robots. Minds Mach. 29, 109–125. 10.1007/s11023-019-09490-x

[B20] de La Mettrie J. O (1747/1960). L'Homme machine, in L'Homme Machine: A Study in the Origins of an Idea, ed Vartanian A. (Princeton, NJ: Princeton University Press), 139–197.

[B21] Descartes R (1664/1985). The philosophical writings of Descartes, in Fourth Meditation: Truth and falsityeds, eds Cottingham J. Stoothoff R. Murdoch D. (Cambridge University Press), 37–43.

[B22] Donati E. Worm M. Mintchev S. van der Wiel M. Benelli G. von der Emde G.. (2016). Investigation of collective behaviour and electrocommunication in the weakly electric fish, mormyrus rume, through a biomimetic robotic dummy fish. Bioinspir. Biomimet. 11:066009. 10.1088/1748-3190/11/6/066009 27906686

[B23] Faria J. J. Dyer J. R. G. Clément R. O. Couzin I. D. Holt N. Ward A. J. W. (2010). A novel method for investigating the collective behaviour of fish: introducing ‘Robofish.' Behav. Ecol. Sociobiol. 64, 1211–1218. 10.1007/s00265-010-0988-y

[B24] Fernández-Juricic E. Gilak N. Mcdonald J. C. Pithia P. Valcarcel A. (2006). A dynamic method to study the transmission of social foraging information in flocks using robots. Anim. Behav. 71, 901–911. 10.1016/j.anbehav.2005.09.008

[B25] Frigg R. (2006). Scientific representation and the semantic view of theories. Theor. 21, 49–65. Available online at: https://www.ehu.eus/ojs/index.php/THEORIA/article/view/553

[B26] Frigg R. Nguyen J. (2017). Models and representation. in Springer Handbook of Model-Based Science, eds Magnani L. Bertolotti T. (Heildelberg; Berlin: Springer International Publishing), 49–102.

[B27] Gergely A. Abdai J. Petró E. Kosztolányi A. Topál J. Miklósi Á. (2015). Dogs rapidly develop socially competent behaviour while interacting with a contingently responding self-propelled object. Anim. Behav. 108, 137–144. 10.1016/j.anbehav.2015.07.024

[B28] Gergely A. Petró E. Topál J. Miklósi Á. (2013). What are you or who are you? The emergence of social interaction between dog and an Unidentified Moving Object (UMO). PLoS ONE 8:e72727. 10.1371/journal.pone.0072727 24015272 PMC3755977

[B29] Giere R. (1990). Explaining Science: A Cognitive Approach. Chicago, IL: University of Chicago Press.

[B30] Göth A. Evans C. S. (2004). Social responses without early experience: Australian brush-turkey chicks use specific visual cues to aggregate with conspecifics. J. Exp. Biol. 207, 2199–2208. 10.1242/jeb.01008 15159424

[B31] Grasso F. W. Consi T. R. Mountain D. C. Atema J. (2000). Biomimetic robot lobster performs chemo-orientation in turbulence using a pair of spatially separated sensors: progress and challenges. Rob. Auton. Syst. 30, 115–131. 10.1016/S0921-8890(99)00068-8

[B32] Gravish N. Lauder G. V. (2018). Robotics-inspired biology. J. Exp. Biol. 221:jeb138438. 10.1242/jeb.138438 29599417

[B33] Gribovskiy A. Halloy J. Deneubourg J.-L. Bleuler H. Mondada F. (2010). Towards mixed societies of chickens and robots. In 2010 IEEE/RSJ International Conference on Intelligent Robots and Systems (IEEE), 4722–28.

[B34] Griparić K. Haus T. Miklić D. Polić M. Bogdan S. (2017). A robotic system for researching social integration in honeybees. PLoS ONE 12:e0181977. 10.1371/journal.pone.0181977 28792955 PMC5549902

[B35] Halloy J. Sempo G. Caprari G. Rivault C. Asadpour M. Tâche F.. (2007). Social integration of robots into groups of cockroaches to control self-organized choices. Science 318, 1155–1158. 10.1126/science.1144259 18006751

[B36] Jolly L. Pittet F. Caudal J.-P. Mouret J.-B. Houdelier C. Lumineau S.. (2016). Animal-to-robot social attachment : initial requisites in a gallinaceous bird. Bioinspir. Biomimet. 11:016007. 10.1088/1748-3190/11/1/016007 26845286

[B37] Kopman V. Polverino G. Laut J. Porfiri M. (2012). Using a bioinspired robotic-fish for closed-loop control of zebrafish response in a preference test, in Proceedings of ASME 2012 5th Annual Dynamic Systems and Control Conference joint with the JSME 2012 11th Motion and Vibration Conference, Vol. 2 (Fort Lauderdale, FL; New York, NY: ASME). 23152102

[B38] Krause J. Winfield A. F. T. Deneubourg J. L. (2011). Interactive robots in experimental biology. Trends Ecol. Evol. 26, 369–375. 10.1016/j.tree.2011.03.015 21496942

[B39] Kubinyi E. Miklósi Á. Kaplan F. Gácsi M. Topál J. Csányi V. (2004). Social behaviour of dogs encountering AIBO, an animal-like robot in a neutral and in a feeding situation. Behav. Processes 65, 231–239. 10.1016/j.beproc.2003.10.003 14998660

[B40] Lambrinos D. Möller R. Labhart T. Pfeifer R. Wehner R. (2000). A mobile robot employing insect strategies for navigation. Rob. Auton. Syst. 30, 39–64. 10.1016/S0921-8890(99)00064-0

[B41] Landgraf T. Bierbach D. Nguyen H. Muggelberg N. Romanczuk P. Krause J. (2016). RoboFish: increased acceptance of interactive robotic fish with realistic eyes and natural motion patterns by live trinidadian guppies. Bioinspir. Biomimet. 11:015001. 10.1088/1748-3190/11/1/015001 26757096

[B42] Landgraf T. Moballegh H. Rojas R. (2008). Design and development of a robotic bee for the analysis of honeybee dance communication. Appl. Bionics Biomech. 5, 157–164. 10.1155/2008/871297

[B43] Landgraf T. Nguyen H. Schroer J. Szengel A. Cl'ement R. J. G. Bierbach D. (2014). Blending in with the shoal: robotic fish swarms for investigating strategies of group formation in guppies, in Living Machines 2014, LNAI, Lecture Notes in Computer Science, eds Duff A. Lepora N. F. Mura A. Prescott T. J. Verschure P. F. M. J. (Cham: Springer International Publishing), 178–189.

[B44] Landgraf T. Oertel M. Kirbach A. Menzel R. Rojas R. (2012). Imitation of the honeybee dance communication system by means of a biomimetic robot, in Living Machines 2012: Biomimetic and Biohybrid Systems, Lecture Notes in Computer Science, eds Prescott T. J. Lepora N. F. Mura A. Verschure P. F. M. J. (Berlin; Heidelberg: Springer Berlin Heidelberg), 132–143.

[B45] Long J. H. (2012). Darwin's Devices: What Evolving Robots Can Teach Us About the History of Life and the Future of Technology. New York, NY: Basic Books.

[B46] Long J. H. Schumacher J. Livingston N. Kemp M. (2006). Four flippers or two? Tetrapodal swimming with an aquatic robot. Bioinspir. Biomim. 1, 20–29. 10.1088/1748-3182/1/1/003 17671301

[B47] Margerie E. De Lumineau S. Houdelier C. Richard Yris M.-A. (2011). Influence of a mobile robot on the spatial behavior of quail chicks. Bioinspir. Biomimet. 6:034001. 10.1088/1748-3182/6/3/034001 21869465

[B48] Marras S. Porfiri M. (2012). Fish and robots swimming together: attraction towards the robot demands biomimetic locomotion. J. R. Soc. Interface 9, 1856–1868. 10.1098/rsif.2012.0084 22356819 PMC3385770

[B49] Martins E. P. Ord T. J. Davenport S. W. (2005). Combining motions into complex displays: playbacks with a robotic lizard. Behav. Ecol. Sociobiol. 58, 351–360. 10.1007/s00265-005-0954-2

[B50] Michelsen A. Bach Andersen B. Storm J. Kirchner W. H. Lindauer M. (1992). How honeybees perceive communication dances, studied by means of a mechanical model. Behav. Ecol. Sociobiol. 30, 143–150. 10.1007/BF00166696

[B51] Mitri S. Wischmann S. Floreano D. Keller L. (2013). Using robots to understand social behaviour. Biol. Rev. 88, 31–39. 10.1111/j.1469-185X.2012.00236.x 22816672

[B52] Ord T. J. Stamps J. A. (2008). Alert signals enhance animal communication in ‘noisy' environments. Proc. Natl. Acad. Sci. U.S.A. 105, 18830–18835. 10.1073/pnas.0807657105 19033197 PMC2596256

[B53] Partan S. R. Larco C. P. Owens M. J. (2009). Wild tree squirrels respond with multisensory enhancement to conspecific robot alarm behaviour. Anim. Behav. 77, 1127–1135. 10.1016/j.anbehav.2008.12.029

[B54] Partan S. R. Otovic P. Price V. L. Brown S. E. (2011). Assessing display variability in wild brown anoles anolis sagrei using a mechanical lizard model. Curr. Zool. 57, 140–152. 10.1093/czoolo/57.2.140

[B55] Patricelli G. L. Coleman S. W. Borgia G. (2006). Male satin bowerbirds, ptilonorhynchus violaceus, adjust their display intensity in response to female startling: an experiment with robotic females. Anim. Behav. 71, 49–59. 10.1016/j.anbehav.2005.03.029

[B56] Patricelli G. L. Uy J. A. C. Walsh G. Borgia G. (2002). Male displays adjusted to female's response. Nature 415, 279–280. 10.1038/415279a 11796996

[B57] Pfeifer R. Lungarella M. Sporns O. (2008). The synthetic approach to embodied cognition, in Handbook of Cognitive Science, eds Calvo P. Gomila A. (Elsevier), 121–137.

[B58] Phamduy P. Polverino G. Fuller R. C. Porfiri M. (2014). Fish and robot dancing together: bluefin killifish females respond differently to the courtship of a robot with varying color morphs. Bioinspir. Biomimet. 9:036021. 10.1088/1748-3182/9/3/036021 25162832

[B59] Polverino G. Abaid N. Kopman V. Macrì S. Porfiri M. (2012). Zebrafish response to robotic fish: preference experiments on isolated individuals and small shoals. Bioinspir. Biomimet. 7:036019. 10.1088/1748-3182/7/3/036019 22677608

[B60] Polverino G. Karakaya M. Spinello C. Soman V. R. Porfiri M. (2019). Behavioural and life-history responses of mosquitofish to biologically inspired and interactive robotic predators. J. R. Soc. Interface 16:20190359 10.1098/rsif.2019.0359 31506048 PMC6769303

[B61] Polverino G. Phamduy P. Porfiri M. (2013). Fish and robots swimming together in a water tunnel: robot color and tail-beat frequency influence fish behavior. PLoS ONE 8, 47–50. 10.1371/journal.pone.0077589 24204882 PMC3808421

[B62] Polverino G. Porfiri M. (2013a). Mosquitofish (Gambusia affinis) responds differentially to a robotic fish of varying swimming depth and aspect ratio. Behav. Brain Res. 250, 133–138. 10.1016/j.bbr.2013.05.008 23684918

[B63] Polverino G. Porfiri M. (2013b). Zebrafish (Danio Rerio) behavioural response to bioinspired robotic fish and mosquitofish (Gambusia affinis). Bioinspir. Biomimet. 8:044001. 10.1088/1748-3182/8/4/044001 23999758

[B64] Powell D. L. Rosenthal G. G. (2017). What artifice can and cannot tell us about animal behavior. Curr. Zool. 63, 21–26. 10.1093/cz/zow091 29491959 PMC5804151

[B65] Reaney L. T. (2009). Female preference for male phenotypic traits in a fiddler crab: do females use absolute or comparative evaluation? Anim. Behav. 77, 139–143. 10.1016/j.anbehav.2008.09.019

[B66] Reaney L. T. Sims R. A. Sims S. W. M. Jennions M. D. Backwell P. R. Y. (2008). Experiments with robots explain synchronized courtship in fiddler crabs. Curr. Biol. 18, 62–63. 10.1016/j.cub.2007.11.047 18211839

[B67] Reeve R. Webb B. Horchler A. Indiveri G. Quinn R. (2005). New technologies for testing a model of cricket phonotaxis on an outdoor robot. Rob. Auton. Syst. 51, 41–54. 10.1016/j.robot.2004.08.010

[B68] Riskin J. (2016). The Restless Clock. A History of the Centuries-Long Argument over What Makes Living Things Tick. Chicago, IL: University of Chicago Press.

[B69] Romano D. Benelli G. Donati E. Remorini D. Canale A. Stefanini C. (2017). Multiple cues produced by a robotic fish modulate aggressive behaviour in siamese fighting fishes. Nat. Sci. Rep. 7, 1–11. 10.1038/s41598-017-04840-0 28680126 PMC5498610

[B70] Romano D. Benelli G. Stefanini C. (2019a). Encoding lateralization of jump kinematics and eye use in a locust via bio-robotic artifacts. J. Exp. Biol. 222:jeb187427. 10.1242/jeb.187427 30446536

[B71] Romano D. Bloemberg J. Tannous M. Stefanini C. (2020a). Impact of aging and cognitive mechanisms on high-speed motor activation patterns: evidence from an orthoptera-robot interaction. IEEE Transac. Med. Robot. Bionics 14, 1–1. 10.1109/TMRB.2020.2977003

[B72] Romano D. Donati E. Benelli G. Stefanini C. (2019b). A review on animal–robot interaction: from bio-hybrid organisms to mixed societies. Biol. Cybern. 113, 201–225. 10.1007/s00422-018-0787-5 30430234

[B73] Romano D. Elayan H. Benelli G. Stefanini C. (2020b). Together we stand-analyzing schooling behavior in naive newborn guppies through biorobotic predators. J. Bionic Eng. 17, 174–184. 10.1007/s42235-020-0014-7

[B74] Ruberto T. Mwaffo V. Singh S. Neri D. Porfiri M. (2016). Zebrafish response to a robotic replica in three dimensions. R. Soc. Open Sci. 3:160505. 10.1098/rsos.160505 27853566 PMC5098991

[B75] Rundus A. S. Owings D. H. Joshi S. S. Chinn E. Giannini N. (2007). Ground squirrels use an infrared signal to deter rattlesnake predation. Proc. Natl. Acad. Sci. U.S.A. 104, 14372–14376. 10.1073/pnas.0702599104 17704254 PMC1950100

[B76] Shi Q. Ishii H. Kinoshita S. Takanishi A. Okabayashi S. Iida N. (2013). Modulation of rat behaviour by using a rat-like robot. Bioinspir. Biomimet. 8:046002 10.1088/1748-3182/8/4/046002 24091776

[B77] Shi Q. Ishii H. Tanaka K. Sugahara Y. Takanishi A. Okabayashi S.. (2015). Behavior modulation of rats to a robotic rat in multi-rat interaction. Bioinspir. Biomimet. 10:056011. 10.1088/1748-3190/10/5/056011 26414400

[B78] Spinello C. Macrì S. Porfiri M. (2013). Acute ethanol administration affects zebrafish preference for a biologically inspired robot. Alcohol 47, 391–398. 10.1016/j.alcohol.2013.04.003 23725654

[B79] Suárez M. (2004). An inferential conception of scientific representation. Philos. Sci. 71, 767–779. 10.1086/421415

[B80] Swoyer C. (1991). Structural representation and surrogative reasoning. Synthese 87, 449–508. 10.1007/BF00499820

[B81] Taylor R. C. Klein B. A. Stein J. Ryan M. J. (2008). Faux frogs: multimodal signalling and the value of robotics in animal behaviour. Anim. Behav. 76, 1089–1097. 10.1016/j.anbehav.2008.01.031

[B82] Toon A. (2012). Similarity and scientific representation. Int. Stud. Philos. Sci. 26, 241–257. 10.1080/02698595.2012.731730

[B83] Webb B. (2001). Can robots make good models of biological behaviour? Behav. Brain Sci. 24, 1033–1050. 10.1017/S0140525X01000127 12412325

[B84] Webb B. (2002). Robots in invertebrate neuroscience. Nature 417, 359–363. 10.1038/417359a 12015617

[B85] Webb B. (2006). Validating biorobotic models. J. Neural Eng. 3, R25–R35. 10.1088/1741-2560/3/3/R01 16921200

[B86] Webb B. Consi T. R. (eds.). (2001). Biorobotics: Methods and Applications. Cambridge, UK: The MIT Press.

[B87] Weisberg M. (2015). Simulation and Similarity: Using Models to Understand the World. Oxford, UK: Oxford University Press.

[B88] Woodward J. (2000). Explanation and invariance in the special sciences. Br. J. Philos. Sci. 51, 197–254. 10.1093/bjps/51.2.197

[B89] Worm M. Landgraf T. Nguyen H. von der Emde G. (2014). Electro-communicating dummy fish initiate group behavior in the weakly electric fish Mormyrus rume, in Living Machines 2014, LNAI, Vol. 8608 LNAI, eds Duff A. Lepora N. F. Mura A. Prescott T. J. Verschure P. F. M. J. (Cham; Heidelberg; New York, NY; Dordrecht; London: Springer), 446–448. 10.1007/978-3-319-09435-9

